# Deep Brain Stimulation for Obsessive-Compulsive Disorder: A Meta-Analysis of Treatment Outcome and Predictors of Response

**DOI:** 10.1371/journal.pone.0133591

**Published:** 2015-07-24

**Authors:** Pino Alonso, Daniel Cuadras, Loes Gabriëls, Damiaan Denys, Wayne Goodman, Ben D. Greenberg, Fiacro Jimenez-Ponce, Jens Kuhn, Doris Lenartz, Luc Mallet, Bart Nuttin, Eva Real, Cinto Segalas, Rick Schuurman, Sophie Tezenas du Montcel, Jose M. Menchon

**Affiliations:** 1 OCD Clinical and Research Unit, Department of Psychiatry, Hospital de Bellvitge, Barcelona, Spain; 2 Bellvitge Biomedical Research Institute-IDIBELL, Barcelona, Spain; 3 CIBERSAM (Centro de Investigación en Red de Salud Mental), Carlos III Health Institute, Barcelona, Spain; 4 Department of Clinical Sciences, Bellvitge Campus, University of Barcelona, Barcelona, Spain; 5 Methodological and Statistical Assessment Unit, Parc Sanitari Sant Joan de Déu—Fundació Sant Joan de Déu, Barcelona, Spain; 6 University Centre for OCD, Department of Psychiatry, UPC-KULeuven, Leuven, Belgium; 7 Department of Psychiatry, Academic Medical Center, University of Amsterdam, Amsterdam, The Netherlands; 8 Brain Imaging Center, Academic Medical Center, University of Amsterdam, and the Netherlands Institute for Neuroscience, Institute of the Royal Netherlands Academy of Arts and Sciences, Amsterdam, The Netherlands; 9 Department of Psychiatry and Behavioral Health System, Icahn School of Medicine at Mount Sinai, New York, New York, United States of America; 10 Department of Psychiatry and Human Behavior, Alpert Medical School of Brown University, Butler Hospital, Providence, Rhode Island, United States of America; 11 Unit of Stereotactic, Functional Neurosurgery and Radiosurgery, General Hospital of Mexico, Mexico City, Mexico; 12 Department of Psychiatry and Psychotherapy, University of Cologne, Cologne, Germany; 13 UPMC-Inserm U1127-CNRS UMR7225, ICM–Brain & Spine Institute, Paris, France; 14 Department of Neurosurgery, UZ Leuven, KU Leuven, Belgium; 15 Department of Neurosurgery, Academic Medical Center, University of Amsterdam, Amsterdam, The Netherlands; 16 UPMC Univ Paris 06, ER4, Modelling in Clinical Research, Paris, France; 17 AP-HP, Hopitaux Universitaires Pitié-Salpétrière Charles-Foix, Department of Biostatistics and Medical Informatics, Paris, France; INSERM / CNRS, FRANCE

## Abstract

**Background:**

Deep brain stimulation (DBS) has been proposed as an alternative to ablative neurosurgery for severe treatment-resistant Obsessive-Compulsive Disorder (OCD), although with partially discrepant results probably related to differences in anatomical targetting and stimulation conditions. We sought to determine the efficacy and tolerability of DBS in OCD and the existence of clinical predictors of response using meta-analysis.

**Methods:**

We searched the literature on DBS for OCD from 1999 through January 2014 using PubMed/MEDLINE and PsycINFO. We performed fixed and random-effect meta-analysis with score changes (pre-post DBS) on the Yale-Brown Obsessive Compulsive Scale (Y-BOCS) as the primary-outcome measure, and the number of responders to treatment, quality of life and acceptability as secondary measures.

**Findings:**

Thirty-one studies involving 116 subjects were identified. Eighty-three subjects were implanted in striatal areas—anterior limb of the internal capsule, ventral capsule and ventral striatum, nucleus accumbens and ventral caudate—27 in the subthalamic nucleus and six in the inferior thalamic peduncle. Global percentage of Y-BOCS reduction was estimated at 45.1% and global percentage of responders at 60.0%. Better response was associated with older age at OCD onset and presence of sexual/religious obsessions and compulsions. No significant differences were detected in efficacy between targets. Five patients dropped out, but adverse effects were generally reported as mild, transient and reversible.

**Conclusions:**

Our analysis confirms that DBS constitutes a valid alternative to lesional surgery for severe, therapy-refractory OCD patients. Well-controlled, randomized studies with larger samples are needed to establish the optimal targeting and stimulation conditions and to extend the analysis of clinical predictors of outcome.

## Introduction

Obsessive-compulsive disorder (OCD) is characterized by the presence of upsetting, persistent thoughts, images, or impulses, which are experienced as intrusive and senseless (obsessions) and/or excessive repetitive behaviors or mental acts (compulsions) intended to neutralize the anxiety induced by the obsessions [1]. OCD has a lifetime prevalence of 2.3% [2] and causes substantial dysfunction in social adjustment, employment, marriage, family relationships and socioeconomic status [3]. Despite exhaustive use of optimal behavioral and pharmacological treatments, an estimated 10% of OCD patients remain resistant to all therapies and suffer from severe symptoms leading to marked functional impairment [4]. Deep brain stimulation (DBS) has been proposed as a last-resort option and an alternative to stereotactic lesional neurosurgery for this group of extremely disabled patients. DBS permits focal, adjustable and reversible neuromodulation through the implantation of electrodes that send electrical impulses to specific locations in the brain. In recent years DBS has been tested as a therapeutic option for several neuropsychiatric conditions including OCD, depression, anorexia nervosa and addictions [5]. In the case of OCD, the therapeutic effect of DBS has been tentatively related to its capacity to modulate abnormal activity and synaptic connectivity in circuits involving the orbitofrontal cortex (OFC), anterior cingulate cortex (ACC) and striatum [6], brain areas that have been implicated in the pathophysiology of the disorder [7]. Reductions in OCD severity in response to DBS range from 52–54% in patients receiving ventral capsule/ventral striatum (VC/VS) or nucleus accumbens (NA) stimulation to 41% in those with electrodes implanted in the subthalamic nucleus (STN) [8]. Percentage of responders–subjects with a reduction in their symptom severity of at least 35%–varies from 10% [9] to 61.5% [10]. These discrepant results may be at least partially related to the differences in anatomical targeting, electrode design and stimulation protocols used. Certain data also suggest that some manifestations of the disorder, i.e. “just-right” experiences or the need for symmetry, may be less likely to respond to DBS [11, 12], although the low number of patients included in each study has complicated the identification of clinical markers of response. In view of the clinical heterogeneity of the disorder, the analysis of these predictors would be extremely helpful to facilitate the selection of candidates for DBS since the technique is not free from potentially severe adverse effects and is a highly economical and human resources requesting option.

So, the goal of the current meta-analysis was 1) to systematically record the treatment effects of DBS in severe therapy-refractory OCD patients, and 2) to identify any clinical variables associated with a better response to this therapeutic approach.

## Material and Methods

### Search strategy for identification of studies

We performed a comprehensive PubMed/MEDLINE and PsycINFO search from January 1999 through January 30, 2014, including the following terms: “deep brain stimulation” or “DBS” in association with “obsessive-compulsive”, “obsessive-compulsive disorder” or “OCD”. These words were searched as key words, title, abstract and Medical Subject Headings. Reference lists from retrieved reports were reviewed for additional relevant studies.

### Selection of studies

Candidate studies–judged on the basis of their title and abstract–had to satisfy the following criteria to be eligible for inclusion in this review: 1) human studies assessing the efficacy of DBS on OCD according to changes on the Yale-Brown Obsessive Compulsive Scale (Y-BOCS) scores or percentage of responders defined by standardized criteria; 2) subjects aged 18–75 years with a diagnosis of OCD according to the Diagnostic and Statistical Manual of Mental Disorders IV [1] or International Classification of Diseases criteria [13]; 3) studies published in English in peer-reviewed journals.

Exclusion criteria:

- reviews of DBS use for OCD not providing results from de novo patients
- discussions of ethical issues related to DBS
- articles focused on biological correlates of DBS use in OCD–neuroimaging, electrophysiological or neuropsychological changes after DBS
- articles focused on the secondary side effects of chronic DBS use in OCD or effects observed during acute stimulation programming
- articles focused on other indications of DBS different from OCD
- articles focused on neurosurgical issues related to DBS implantation for OCD
- studies on animal models of DBS use in OCD

### Data extraction

Data were recorded as follows:

- sample characteristics: age, gender, age at onset of OCD, duration of OCD, OCD symptom dimensions.
- DBS-related: brain target, lead model, duration of stimulation.
- study-related: single or double-blind; sham-controlled; parallel or crossover designs
- primary outcome measure: score changes (pre-post DBS) on the Y-BOCS
- secondary outcome measure: number of responders to treatment based on standardized criteria (> 35% reduction in post-treatment Y-BOCS scores) and changes on quality of life (QOL) measures
- acceptability of treatment: overall dropout rates and side effects

### Data synthesis and analyses

Analyses were performed using the statistical software R3.0.1 [14] with meta package for meta-analysis [15] and IBM SPSS Version 20 (IBM Corporation, Chicago, IL, USA). Weighted proportion meta-analysis was used to adjust for study size using the DerSimonian-Laird model to allow for heterogeneity inclusion in the analysis. Effect sizes were calculated with fixed and random-effects models, and risk ratios were presented as a forest plot. The forest plot shows study-specific risk ratios (and their 95% CIs) and the relative weighted contribution of each study, as well as the risk ratio estimate pooled across all studies. Heterogeneity was assessed using the *Q* statistics and *I* ^*2*^ index [16]. Values of p< 0.1 for the former and > 35% for the latter were deemed as indicative of between-study heterogeneity [17]. Student’s “t” test and Spearman’s rank correlation were used to analyze the association of age and age at OCD onset with response to DBS. Pearson’s chi squared test and Wilcoxon test were used to study gender differences and influence of neuroanatomical target on response to DBS. Finally, Fisher’s exact test was used to assess differences on response to DBS according to OCD symptom dimensions.

## Results

### Literature search

**Fig 1:**
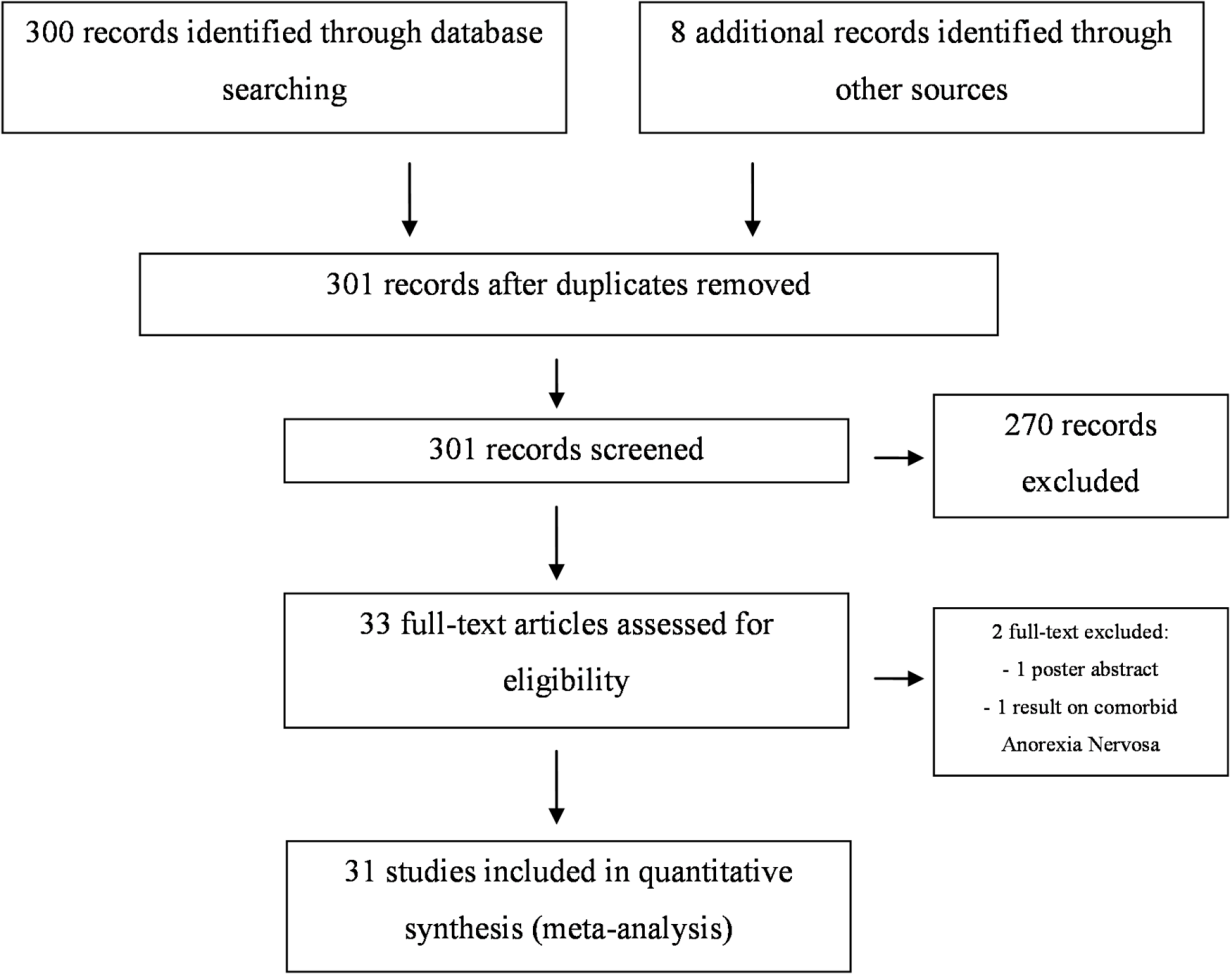
Flow of information according to PRISMA statement, study selection and reasons for exclusion.

Flow of information according to PRISMA statement, study selection and reasons for exclusion are provided in Fig 1. Our electronic and reference list search found 301 studies, after discarding duplicates, that were potentially relevant to this meta-analysis. Of these, 270 were not included in the analysis because they met exclusion criteria. Thirty-three articles met the eligibility criteria (see Table 1). One was excluded because it was the abstract of a poster presentation [18] and another reported results on comorbid Anorexia Nervosa in an OCD patient treated with DBS but did not provide information on changes in OCD symptoms [19].

**Table 1:** Characteristics of the 31 studies included in the meta-analysis.

Authors	Year	Number of patients	Number of new patients	Final observation period (months)	DBS target	Double blind	% Y-BOCS reduction	Responders (ratio)
Nuttin et al. [20]	1999	1*	1	Unknown	ALIC	Yes	Unknown	1/1
Mallet et al. [21]	2002	2	2	6	Subthalamic nucleus	No	80.7–82.6%	2/2
Gabriels et al. [22]	2003	3	2^a^	15–31	ALIC	2 Yes/1 No	21–50%	1 /2
Nuttin et al. [23]	2003	8	5^b^	21–31	ALIC	2 Yes/3 No	17.6–57.8%	2 /3 ^l^
Anderson & Ahmed [24]	2003	1	1	3	ALIC	No	79.4%	1/1
Sturm et al. [25]	2003	4	4	24–30	Nucleus accumbens	No	Unknown	3 /4
Fontaine et al. [26]	2004	1	1	12	Subthalamic nucleus	No	96.8%	1/1
Aouizerate et al. [27]	2004	1	1	27	Ventral caudate nucleus	No	52.0%	1/1
Aouizerate et al. [28]	2005	1	0	27	Ventral caudate nucleus	No		
Abelson et al. [29]	2005	4	4	4–23	ALIC	Yes	0–73.3%	2/4
Greenberg et al. [12]	2006	10	10	6–36	ALIC	No	35.5% ^j^	4/8 ^m^ ^,n^
Kuhn et al. [30]	2007	1	1	30	Nucleus accumbens	No	52%	1/1
Plewnia [31]	2008	1	1	12	ALIC	No	Unknown	1/1
Guehl et al. [32]	2008	3	2^d^	12	Ventral caudate nucleus	No	52–71.4%	2/2
Mallet et al. [33]	2008	16	16	3	Subthalamic nucleus	Yes	37.8%	7/16
Aouizerate et al. [34]	2009	2	0	30	Ventral caudate nucleus	No		
Jiménez-Ponce et al. [35]	2009	5	5	12–36	Inferior thalamic peduncle	No	40–58.3%	5/5
Servello et al. [36]	2009	4	4	9–19	ALIC	No	9.0–60.5%	2/4
Huff et al. [9]	2010	10	10	12	Nucleus accumbens	Yes	0–55.5%	1/10
Greenberg et al. [10]	2010	26	8^f^	3–36	VC/VS	Yes	0–62.1%	3/5 ^o^
Goodman et al. [37]	2010	6	1^g^	12	VC/VS	Yes	91.3%	1/1
Burdick et al. [38]	2010	1	0^h^	30	ALIC	Yes		
Denys et al. [11]	2010	16	16	21	Nucleus accumbens	Yes	46% ^j^	9/16 ^m^
Franzini et al. [39]	2010	2	2	24–27	Nucleus accumbens	No	33.3–44.7%	1 /2
Piallat et al. [40]	2011	9	6^i^	Unknown	Subthalamic nucleus	Unknown	Unknown	Unknown
Tsai et al. [41]	2012	4	4	15–21	VC/VS	No	0–70.5%	2/4
Chabardes et al. [42]	2012	4	4	6	Subthalamic nucleus	No	34.3–72.4%	1 /2
Jimenez-Ponce et al. [43]	2012	6	1^k^	36	Inferior thalamic peduncle	No	82.5%	1/1
Roh et al. [44]	2012	4	4	24	VC/VS		45.7–61.1%	4/4
Grant et al. [45]	2012	1	1	8	Nucleus accumbens	No	68.7%	1/1
Sachdev et al. [46]	2012	1	1	8	Nucleus accumbens	No	90%	1/1
Total			116					

ALIC: Anterior limb of the internal capsule

VC/VS: Anterior limb of the internal capsule–ventral capsule- and ventral striatum

Response defined as a reduction of Y-BOCS scores > 35%

* The authors make reference to four patients with severe OCD treated with DBS but information is provided just for one.

^a^ One patients included in Nuttin et al., 1999 [20]

^b^ Three patients included in Gabriels et al., 2003 [22]

^c^ Patient included in Aouizerate et al., 2004 [27]

^d^ One patient included in Aouizerate et al., 2004 [27]

^e^ Patients included in Guehl et al., 2008 [32]

^f^ Ten patients included in Greenberg et al., 2006 [12] and five patients included in Nuttin et al., 2003 [23]

^g^ Five patients included in Greenberg et al., 2010 [10]

^h^ Patient included in Goodman et al., 2010 [37]

^i^ Three patients included in Mallet et al., 2008 [33]

^j^ Two patients included in Mallet et al., 2008 [33]

^k^ Five patients included in Jimenez-Ponce et al., 2009 [35]

^l^ Information on long term response available for 3 patients

^m^ Individual data not available, results correspond to mean results from the study

^n^ Information available in 8 patients

^o^ Information available in 5 patients

### Studies included: main characteristics

Thirty-one studies were included in this meta-analysis [9–12, 20–46], comprising 116 subjects with OCD treated with DBS (S1 Table). The main characteristics of the studies included are described in Table 1. Twenty-four studies including 83 patients addressed DBS of “striatal areas”, including the anterior limb of the internal capsule (ALIC), the ventral capsule and ventral striatum (VC/VS), the nucleus accumbens (NA) or the ventral caudate nucleus; five studies including 27 patients reported results on stimulation of the subthalamic nucleus, and two studies from Mexico, including six patients, described results of DBS applied at the inferior thalamic peduncle. Stimulation parameters were highly heterogeneous between studies: although all them employed high frequency stimulation (from 100 to 130 Hz), pulse width ranged from 60 to 450 μs and voltage from 2 to 10,5 V; different models of electrodes (3387, 3887, 3487; Medtronic Inc, Minneapolis, Minnesota) as well as active contact points were also used in the different samples. Authors of some of the articles included in the meta-analysis were contacted to gather further information.

### Pre-post severity of OCD symptoms

**Fig 2:**
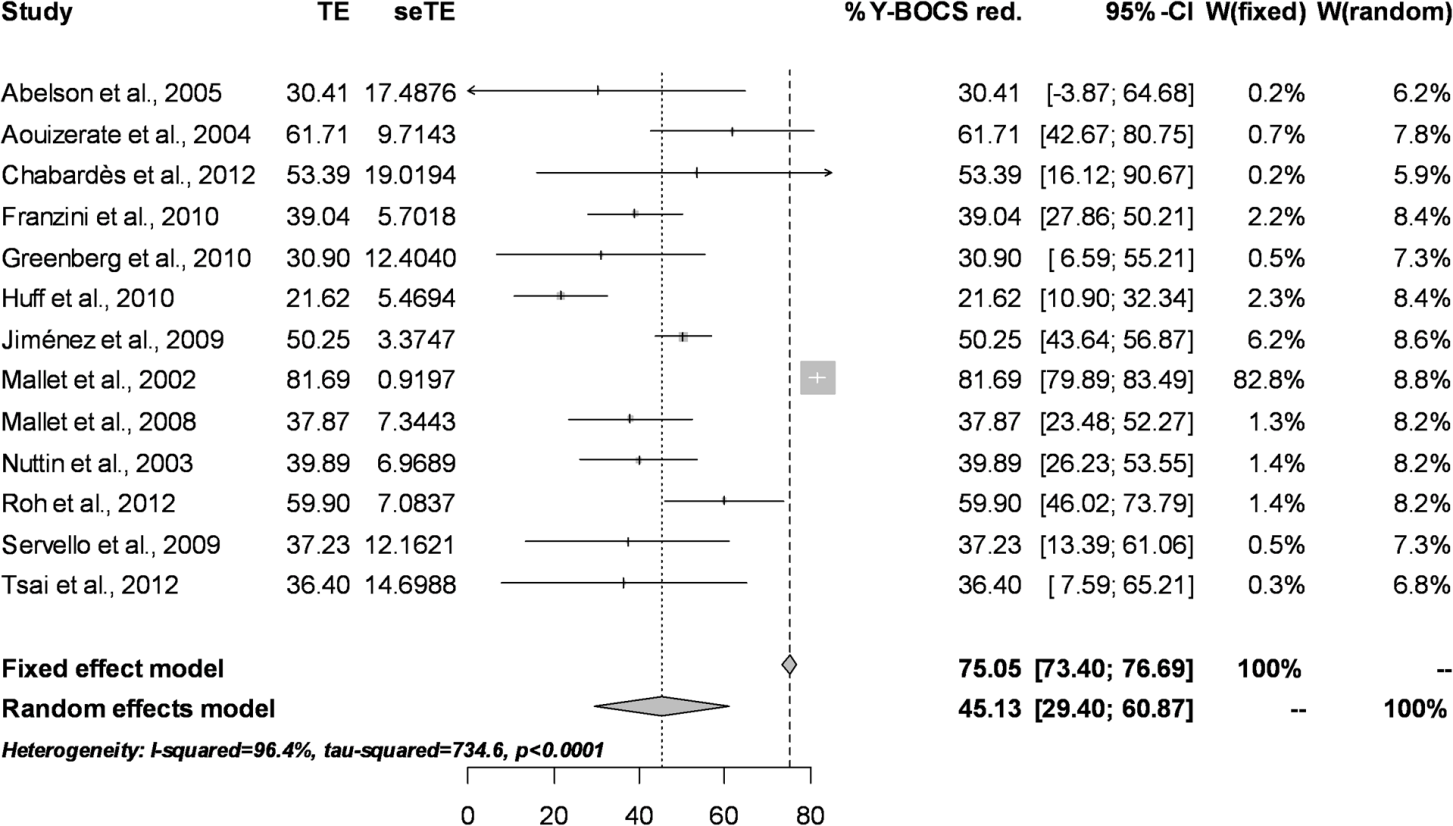
Forest Plot for percentage of improvement in Y-BOCS scores.

Patient-level data relating to Y-BOCS score changes were available for 13 studies, including 66 patients. Mean percentages of reduction, standard deviation and standard error for each study were calculated to perform the meta-analysis on the percentage of improvement. The fixed effect model could not be used since it overestimates the percentage of improvement due to the excessive weight of Mallet et al.’s results [21] in two patients with high and almost identical percentages of improvement. The random effect model estimates the global percentage of improvement at 45.1% (95% CI = 29.4% to 60.8%). This wide confidence interval can be attributed to the reduced sample size of the studies as well as to their heterogeneity (Q = 734.6, df = 12, p <0.0001; *I* ^*2*^ = 96.4%) (see Fig 2 for the associated Forest Plot)

### Percentage of responders

**Fig 3:**
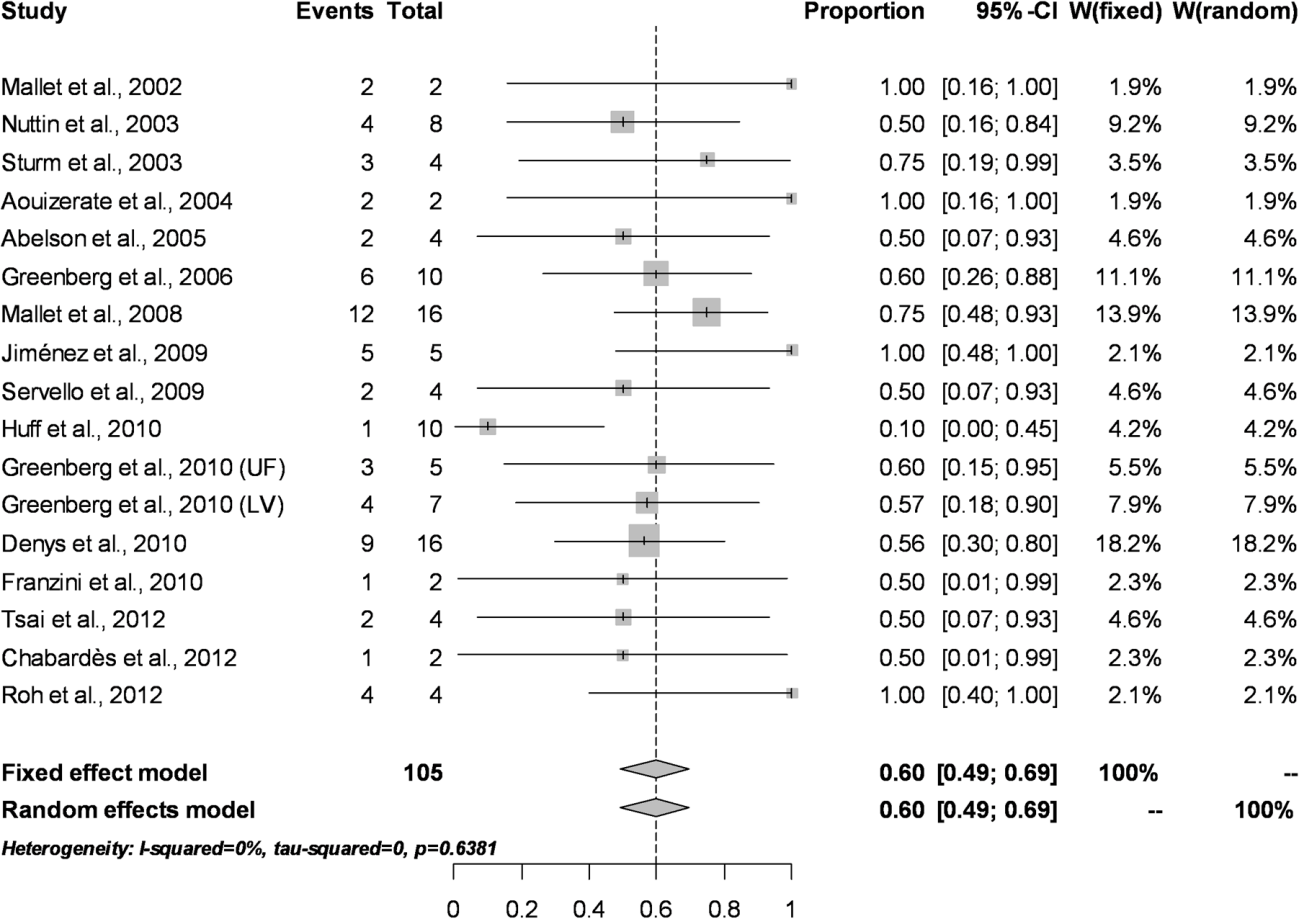
Forest Plot for percentage of responders according to standardized criteria (> 35% reduction in post-treatment Y-BOCS scores).

Response to treatment–defined by operationalized criteria as a reduction on Y-BOCS scores > 35%–was analyzed in studies including more than one subject in order to estimate its variability. Patient-level data was available from 12 studies, while four provided results on pooled data (percentage of responders in the study). A fixed effect model estimated the global percentage of responders at 60.0% (95% CI = 49.0% to 69.0%). The random effect model detected identical results due to the low sample size of the studies. Results for *Q* statistics (Q = 13.47, df = 15, p = 0.63) and *I* ^*2*^ index (*I* ^*2*^ = 0%) suggested that the low sample sizes did not permit a correct estimation of heterogeneity (see Fig 3 for the associated Forest Plot).

### Quality of life

Information on the impact of DBS on quality of life was available for 29 patients [9, 37, 47]. Goodman et al., [37] described a significant increase of the Medical Outcomes Study Short Form Health Survey (SF-36) vitality score after one year of chronic stimulation of the anterior limb of the internal capsule and adjacent ventral striatum. Similarly, Huff et al., [9] reported a significant improvement of quality of life assessed through the Modular System of Quality of Life (MSLQ) (from 41.3 ± 15.8 to 53.2 ± 19.8) in a group of 9 patients stimulated at VC/VS for twelve months. Finally, Ooms et al., [47] detected a significant improvement of 90% over time in the general WHO Quality of Life Scale-Brief Version score (WHOQOL-BREF) as well as in the physical (39.5%), psychological (39.5%) and environmental (16%) domains of the scale in 13 of the 16 patients included in Denys et al’s study who underwent DBS of the nucleus accumbens for 3 to 5 years.

### Clinical predictors of response

#### DBS targets

Patient-level data were available for 45 patients who received DBS in striatal areas–including VC/VS, ALIC, NAc and NC–and 21 patients who were implanted in the STN. No significant differences were detected between the two targets in terms of the percentage of reduction in Y-BOCS scores (striatal: 39.0% ± 25.2 versus STN: 46.3% ± 31.5, t: -1.0, df = 64, p-value = 0.3) or in percentage of responders (striatal: 55.5% versus STN: 52.3%, χ^2^ = 0.06, p = 0.8).

#### Age and gender

No significant differences were detected between responders and non-responders to DBS in terms of age (responders 38.6 years ± 11.1 versus non-responders 37.2 years ± 8.4, t: -0.6, df = 74.9, p-value = 0.5) or gender (responders: 26/19 male/female; non-responders: 20/14 male/female, χ^2^ = 0.009, p = 0.9). Current age was not correlated with percentage of Y-BOCS score reduction (Spearman’s Rho = 0.07, p = 0.5). No significant differences were detected between males and females in percentage of Y-BOCS score reduction (male: 41.7% ± 27.1 versus female: 43.4% ± 27.0, Wilcoxon test W = 272.5, p = 0.2) (S2 Table).

#### Age at OCD onset

Responders to DBS reported a significantly older age at onset of OCD than non-responding patients (responders 17.1 years ± 7.9 vs non-responders 13.7 years ± 6.9, t = -2.0, df = 67.1, p = 0.04, 95% CI = -6.7 to -0.03). A tendency was detected for a significant positive correlation between age at onset of OCD and percentage of Y-BOCS scores reduction after DBS (Spearman’s Rho = 0.2, p = 0.05).

No significant differences in years of OCD duration prior to DBS implantation were detected between responders and non-responders (responders 20.5 years ± 11.1 vs non-responders 23.8 years ± 9.1, t = 1.3, df = 68.9, p = 0.1).

#### OCD symptom dimensions

No significant differences between responders and non-responders to DBS were detected for the presence of aggressive/checking symptoms (responders: 42.5% vs non-responders: 31.2%, Fisher’s Exact test OR = 3.2, p = 0.1, 95% CI = 0.5 to 34.8), contamination/cleaning (responders: 45.1% vs non-responders: 59.3%, Fisher’s Exact test OR = 1.4, p = 0.7, 95% CI = 0.3 to 6.9), symmetry/ordering (responders: 30.1% vs non-responders: 34.3%, Fisher’s Exact test OR = 0.5, p = 0.5, 95% CI = 0.1 to 2.4), hoarding (responders: 7.5% vs non-responders: 3.1%, Fisher’s Exact test OR = 1.3, p = 1, 95% CI = 0.09 to 78.6), and somatic obsessions and compulsions (responders: 15.6% vs non-responders: 7.1%, Fisher’s Exact test OR = 3.2, p = 0.1, 95% CI = 0.5 to 34.8).

Responders more frequently reported obsessions and compulsions of sexual/religious content than non-responders (responders: 33.0% vs non-responders: 0%, p = 0.009) (S2 Table).

### Acceptability of treatment

Five patients dropped out from DBS without completing the planned period of stimulation, representing 4.7% of the implanted patients. Two of these cases were in the early study by Nuttin et al. [23]; both patients finally underwent anterior capsulotomy due to the limited benefits of DBS and extremely fast battery depletion. The other three subjects were from the Mexican group who received DBS at the inferior thalamic peduncle [35]. One died of a cocaine overdose, another presented tuberculous meningitis and was explanted, and the last one stopped attending follow-up controls after 18 months of DBS. Side effects reported in the various studies are presented in Table 2.

**Table 2:** Adverse effects of DBS reported in the studies included in the meta-analysis.

ADVERSE EVENT	n	%
Surgery related		
Intracerebral hemorrhage	3	2.6
Wound infection	5	4.3
Headache	7	6.0
Tonico-clonic seizure	1	0.9
Scalp tingling or numbness	7	6.0
Device related		
Feeling of extension leads, mainly in neck and ear area	10	8.6
Feeling of neurostimulator in chest or abdomen	2	1.7
Break in a stimulating lead or an extension wire	3	2.6
Stimulation related		
Hypomanic symptoms	23	19.8
Disinhibition*	7	6.0
Transient confussion	1	0.9
Stomachache, dizziness, nausea	7	6.0
Enuresis	3	2.6
Olfactory perceptions	4	3.4
Paresthesias, tingling	4	3.4
Tightness at jaw area	2	1.7
Diplopia	1	0.9
Weight gain	5	4.3
Weight loss	1	0.9
Insomnia	4	3.4
Forgetfulness, difficulty findings words, memory complains	9	7.8
Anxiety worsening	25	21.6
Panic attacks	1	0.9
Throbbing, flushing**	12	10.4
Depressive mood	5	4.3
Suicidal ideation	4	3.4
Impulsivity	2	1.7
Speech disturbances	2	1.7

*Not fullfilling criteria for hypomanic episode

** Without other symptoms of a panick attack

## Discussion

The aim of this study was to measure the response to DBS in severe treatment-resistant OCD patients using meta-analysis. The data available from 116 subjects produced a global percentage of Y-BOCS score reduction of 45.1% and a global percentage of responders of 60.0%. Better response to DBS was associated with older age at OCD onset and with the presence of sexual/religious obsessions and compulsions. No significant differences were detected in the percentage of responders or in Y-BOCS score reduction between patients who received stimulation of striatal areas and those with STN implanted electrodes.

These results confirm that DBS appears to have an efficacy comparable to that reported for capsulotomy or cingulotomy, ablative techniques after which 64% and 56% of patients respectively are rated as significantly improved [48, 49]. Nevertheless, severe adverse effects seem to be less frequent with DBS than with lesional neurosurgery. Three cases of intracranial hemorrhages were reported, representing 2.6% of the total number of patients, compared with figures of 15.8% in some studies of ablative interventions [50]. Five subjects presented an infection of the scalp, chest or abdominal wound, but they were controlled with antibiotic therapy, and just one patient suffered a tonic-clonic seizure. Interestingly, no persistent frontal syndrome, cognitive impairment or personality changes have been described for OCD patients receiving DBS. The most frequent stimulation-related adverse effect was a hypomanic state or at least some kind of mood disinhibition, reported in nearly one from five patients. Transient worsening of anxiety while searching for optimal stimulation parameters has also been frequently described. Nevertheless, almost all studies describe these stimulation-related adverse effects as mild, transient and reversible after the adjustment of the stimulation parameters. Five drop-outs were registered among the 116 implanted patients worldwide. Two of them were patients in the early study in Belgium by Nuttin et al. [23], when experience in the use of the technique was still limited, while the last three were from the Mexican group implanted at the inferior thalamic peduncle [35]. This Mexican sample is not obviously comparable to others included in this meta-analysis, since 50% of the subjects presented alcohol and cocaine dependence, a comorbidity generally considered as an exclusion criterion for DBS use in OCD. So DBS, although not an innocuous procedure, appears to constitute a safe therapeutic option for severe treatment-resistant OCD patients, associated with mild and transient emotional and somatic side effects. On the other hand, DBS imposes its own burdens including need for programming by an expert center, battery depletion, device failures, need for urgent interventions in the event of an emergent DBS-related side effect and high economic cost.

Most published studies focus their attention on symptom reduction after DBS and scarce data is available for changes on quality of life in these highly-resistant and chronic severally ill patients [9, 37, 47]. Although results are not easily comparable because of the heterogeneity of the assessment tools, studies suggest that despite the invasive nature of the treatment and the discomfort derived from the surgical procedure and the stimulation process, most patients report a significant improvement in at least some aspects of their quality of life. Interestingly, this improvement was not directly correlated with the reduction of symptom severity and was reported even by non-responding patients. Moreover QOL keep on improving years after DBS initiation, even when no further reduction of OCD severity was evident, suggesting than factors other than OCD intensity–anxiety release, reward processing and motivation, affective status- influence QOL and that patients need time to adapt to and benefit of their new situation.

Distant DBS effects on abnormal neural connectivity in the cortico-striato-thalamo-cortical circuit involved in OCD might explain why stimulation of different brain regions finally achieves similar percentages of improvement. Stimulation of STN has been reported to decrease OFC and mPFC metabolism as well as ACC activity [51] while stimulation of the ALIC has similarly been associated with decreased OFC [23, 29], subgenual ACC and right DLPFC metabolism [52]. Interestingly, while STN stimulation did not significantly modify comorbid depressive and anxiety symptoms [33], a significant and early improvement in mood and anxiety levels, preceding any change in OCD severity, is commonly reported in patients receiving stimulation in striatal areas [11]. Future studies should address the local and distant effects responsible for mutual as well as distinct mechanisms of action of DBS depending on specific targets in order to personalize the choice of the optimal implantation area according to the individual presentation of the illness.

Better response to DBS was associated with older age at OCD onset. Age of onset has been postulated as an important marker for subtyping OCD. Early age of onset patients show more severe forms of OCD, poorer prognosis for pharmacological treatment, higher familial aggregation of both OCD and tic disorders, and a higher specific comorbidity pattern mainly with ADHD, Tourette’s syndrome and bipolar disorder [53, 54]. A few studies have directly addressed the existence of differences in neuroimaging findings between OCD patients with early and late onset of the disorder, with inconclusive results. Pediatric studies suggest that children and adolescents with OCD show abnormalities of the putamen, globus pallidus and thalamus [55]. A recent study by Correia et al. [56] addressing the concentration of iron in the basal ganglia suggested a neurobiological distinction between early and late onset OCD: late onset patients, but not early onset ones, showed significantly higher iron concentrations than healthy controls, particularly in this area, although it is not clear whether iron metabolism plays a direct role in OCD or is just a correlation of other dysfunctions such as serotonergic neurotransmission. Therefore, further studies are needed to determine whether any specific structural or functional brain difference associated with early-onset OCD mediates its poorer response to DBS.

According to the results of this meta-analysis, the presence of sexual/religious obsessions and compulsions was associated with a significantly better response to DBS. Recent studies have associated this OCD clinical dimension with specific brain functional connectivity patterns: patients with more sexual/religious obsessions demonstrated relatively greater connectivity between the ventral caudate and the middle and anterobasal insular cortex than patients with other symptom dimensions as well as healthy controls in a study addressing alterations of ventral corticostriatal functional connectivity in OCD [57]. Since Figee et al. [58] recently reported that the reduction of OCD symptoms after DBS was correlated with a fall in excessive frontostriatal connectivity recorded at baseline, it might be hypothesized that abnormal insulo-striatal connectivity is especially sensitive to the capacity of DBS to normalize brain connectivity. Further neuroimaging studies focusing on changes in connectivity patterns in relation to the response to DBS of different OCD symptom dimensions are needed to confirm this hypothesis.

The present manuscript has a number of limitations. First, the small sample sizes in the studies included complicate the assessment of inter-study heterogeneity. The studies were heterogeneous in terms of anatomical targeting, electrode design and stimulation parameters. This makes the comparison between studies difficult, and reflects the fact that DBS for OCD is a tool that is still under development. Second, we decided to address the response to DBS in all available patients worldwide instead of restricting our analysis to double-blind sham-controlled studies, since only six of the published studies (with only 45 subjects) reported this design in their methodology. Moreover, even in this small body of studies, the duration of active and sham periods was not easily comparable since it lasted from minutes [20] to three months [33], including 15 days [11], 21 days [29] or 30 days [37]. Nevertheless, in all these six studies, active stimulation was significantly more effective than the sham condition, which had to be shortened or cancelled in many patients due to severe clinical deterioration. There is a need for further well-controlled randomized trials to compare active versus sham DBS. Third, information on OCD symptom dimension, which emerged as one of the clinical predictors of response, was not assessed using specifically designed tools in any study even though it was available for 95 patients. The information must therefore be extracted from clinical descriptions, which limits its replicability. Fourth, no meta-regression analyses could be conducted to establish predictors of response, owing to the small number of patients included. Statistical comparison of subgroups was used, instead, as an exploratory method to address this important clinical issue. Finally, as in all meta-analyses, a potential publication bias and the risk of including limited-quality trials must be considered. We tried to address these concerns by the comprehensive and systematic review of the literature and the use of stringent inclusion criteria.

Although the number of severe OCD treatment-resistant patients treated with DBS is still low, and optimal targeting and stimulation parameters are still under debate, the results of this meta-analysis confirm that DBS constitutes an alternative to ablative surgery for this group of extremely ill patients and presents an acceptable adverse effect profile. Further well-controlled randomized studies in larger samples are needed to confirm and extend our findings on clinical predictors of response, and thus to improve both patient selection and response rate.

## Supporting Information

S1 Table Sociodemographic and clinical data of 116 patients included in the metaanalysis. (DOC)

S2 Table Comparison of sociodemographic and clinical data between responders and non-responders to DBS*. (DOC)

S1 Text Prisma 2009 checklist. (DOC)
