# Supplementary material for: Deep Brain Stimulation for Obsessive-Compulsive Disorder: A Meta-Analysis of Treatment Outcome and Predictors of Response
Source: PLoS One. 2015 Jul 24;10(7):e0133591. doi: 10.1371/journal.pone.0133591 (PMC4514753; doi:10.1371/journal.pone.0133591)
Supplement: S1 Table — (DOC) [file pone.0133591.s001.doc]

Supplemental Table 1. Sociodemographic and clinical data of 116 patients included in the metaanalysis.

|  |  | Number of patients with available information |
| --- | --- | --- |
| Gender (male/female) | 62/49 | 111 |
| Age (years, mean, SD, range) | 38.6 ± 10.3 (18-65) | 107 |
| Age at OCD onset  (years, mean, SD, range) | 15.6 ± 7.2 (4-35) | 101 |
| Onset before 18 years old  (n, %) | 73 (69.5 %) | 105 |
| Duration of OCD  (years, mean, SD, range) | 22.8 ± 10.4 (5-50) | 101 |
| Y-BOCS baseline  (mean, SD, range) | 33.2 ± 3.9 (23-40) | 106 |
| Y-BOCS final  (mean, SD, range) | 18.8 ± 9.0 (0-39) | 72 |
| Y-BOCS reduction after DBS (%) | 42.5 ± 26.7 (0-100) | 72 |
| Duration of DBS (months) | 16.3 ± 10.3 (3-36) | 103 |
| Responders* (n,%) | 46 (57.5 %) | 80 |
| Symptom dimensions (present, n,%) |  |  |
| Aggressive/checking | 35 (36.8 %) | 95 |
| Contamination/cleaning | 52 (54.7 %) | 95 |
| Symmetry/ordering | 36 (37.8 %) | 95 |
| Sexual/religious | 14 (17.7 %) | 79 |
| Hoarding | 5 (5.3 %) | 95 |
| Somatic | 7 (10.1 %) | 69 |
| Brain target (n,%) |  |  |
| VC/VS+ALIC+NA+NC | 83 (71.6 %) | 110 |
| Subthalamic nucleus | 27 (23.3 %) | 110 |
| Peduncle Thalamic Inferior | 6 (5.2 %) | 110 |

* Response defined as Y-BOCS reduction > 35%.
