# Supplementary material for: Deep Brain Stimulation for Obsessive-Compulsive Disorder: A Meta-Analysis of Treatment Outcome and Predictors of Response
Source: PLoS One. 2015 Jul 24;10(7):e0133591. doi: 10.1371/journal.pone.0133591 (PMC4514753; doi:10.1371/journal.pone.0133591)
Supplement: S2 Table — (DOC) [file pone.0133591.s002.doc]

Supplemental Table 2. Comparison of sociodemographic and clinical data between responders and non-responders to DBS*.

|  | Responders  (n=46) | Non-responders  (n=34) | t | p | 95% Confidence Interval |
| --- | --- | --- | --- | --- | --- |
| Age (years, mean, SD, range) | 38.8 ± 11.1 | 37.2 ± 8.4 | -0.6 | 0.4 | -6.0 3.2 |
| Age at OCD onset  (years, mean, SD, range) | 17.1 ± 7.1 | 13.7 ± 6.9 | -2.0 | **0.03** | -6.7 -0.03 |
| Duration of OCD  (years, mean, SD, range) | 20.5 ± 11.1 | 23.8 ± 9.1 | 1.3 | 0.1 | -1.5 8.1 |
| Y-BOCS baseline  (mean, SD, range) | 33.1 ± 4.3 | 32.7 ± 3.9 | -0.3 | 0.7 | -2.2 1.5 |
| Y-BOCS final  (mean, SD, range) | 12.8 ± 5.7 | 27.1 ± 5.4 | 10.6 | **<0.0001** | 11.6 16.9 |
| Y-BOCS reduction after DBS (%) | 61.2 ± 16.3 | 16.3 ± 12.7 | -12.5 | **<0.0001** | -52.0 -37.7 |
| Duration of DBS (months) | 15.1 ± 10.1 | 11.3 ± 7.6 | -1.7 | 0.07 | -8.0 0.4 |
|  |  |  | Χ^2^ | p |  |
| Gender (male/female) | 26/19 | 20/14 | 0.009 | 0.9 |  |
| Symptom dimensions (present/absent, n,%) |  |  |  |  |  |
| Aggressive/checking | 17/40 (42.5 %) | 10/32 (31.2%) | 0.9 | 0.3 |  |
| Contamination/cleaning | 18/40 (45 %) | 19/32 (59.3%) | 1.4 | 0.2 |  |
| Symmetry/ordering | 12/40 (30 %) | 11/32 (34.3%) | 0.1 | 0.6 |  |
| Sexual/religious | 11/33 (33 %) | 0/23 (0%) | 9.5 | **0.002** |  |
| Hoarding | 3/40 (7.5 %) | 1/32 (3.1%) | 0.6 | 0.4 |  |
| Somatic | 5/32 (15.6 %) | 1/14 (7.1%) | 0.6 | 0.4 |  |
| Brain target (n,%) |  |  |  |  |  |
| VC/VS+ALIC+NA+NC | 29 (63.0 %) | 24 (70.5 %) | 0.06 | 0.8 |  |
| Subthalamic nucleus | 11 (23.4%) | 10 (29.4%) |  |  |  |
| Peduncle Thalamic Inferior | 6 (12.7 %) |  |  |  |  |

* Response defined as Y-BOCS reduction > 35%.
